# A phase 1 study of lirilumab (antibody against killer immunoglobulin-like receptor antibody KIR2D; IPH2102) in patients with solid tumors and hematologic malignancies

**DOI:** 10.18632/oncotarget.24832

**Published:** 2018-04-03

**Authors:** Norbert Vey, Lionel Karlin, Sophie Sadot-Lebouvier, Florence Broussais, Dominique Berton-Rigaud, Jérôme Rey, Aude Charbonnier, Delphine Marie, Pascale André, Carine Paturel, Robert Zerbib, Jaafar Bennouna, Gilles Salles, Anthony Gonçalves

**Affiliations:** ^1^ Institut Paoli-Calmettes, Marseille, France; ^2^ Aix-Marseille Université, Marseille, France; ^3^ Centre Hospitalier Universitaire de Lyon Sud, Service d’Hématologie, Pierre Bénite, France; ^4^ Institut de Cancérologie de l’Ouest–Site René Gauducheau, St Herblain, France; ^5^ Innate Pharma, Marseille, France

**Keywords:** monoclonal antibody, cancer immunotherapy, toxicity, natural killer cells

## Abstract

**Purpose:**

Anti-KIR monoclonal antibodies (mAbs) can enhance the antitumor responses of natural killer (NK) cells. We evaluated the safety of the anti-KIR2D mAb lirilumab in patients with various cancers.

**Experimental design:**

Thirty-seven patients with hematological malignancies (*n* = 22) or solid tumors (*n* = 15) were included in the study. Dose escalation (0.015 to 10 mg/kg) was conducted following a 3 + 3 design. Patients were scheduled to receive four cycles of treatment. In a second (extension) phase 17 patients were treated at 0.015 (*n* = 9) or 3 mg/kg (*n* = 8).

**Results:**

No dose-limiting toxicity was recorded. The most frequent lirilumab-related adverse events were pruritus (19%), asthenia (16%), fatigue (14%), infusion-related reaction (14%), and headache (11%), mostly mild or moderate. Pharmacokinetics was dose-dependent and linear, with minimal accumulation resulting from the 4-weekly repeated administrations. Full KIR occupancy (>95%) was achieved with all dosages, and the duration of occupancy was dose-related. No significant changes were observed in the number or distribution of lymphocyte subpopulations, nor was any reduction in the distribution of KIR2D-positive NK cells.

**Conclusions:**

This phase 1 trial demonstrated the satisfactory safety profile of lirilumab up to doses that enable full and sustained blockade of KIR.

## INTRODUCTION

Recently, antibodies that block the cytotoxic T-lymphocyte-associated protein 4 (CTLA-4) or programmed death-1/programmed death-ligand-1 (PD-1/PD-L-1) immune checkpoints have highlighted the potential of strategies that target tumor-induced inhibition of T cells to induce clinically relevant tumor control [1]. However, many patients fail to respond optimally to PD-1 and CTLA-4 blockers, maybe due to lack of potential immunological targets. Therefore, there is a need for new drugs that block alternative immune checkpoint receptors. The mobilization of additional types of immune effector cells, such as natural killer (NK) cells, which can cooperate with T cells to produce coordinated antitumor responses, may also be a promising therapeutic strategy.

NK cells are critical effectors of the innate immune system; they are regulated by a balance of signaling from activating and inhibitory receptors and possess potent anticancer effects in a variety of tumor models [2, 3]. Therefore, quantitative or functional alterations of NK cells might contribute to cancer progression. For example, NK cell number and function correlate with relapse-free survival in acute myeloid leukemia (AML) [4, 5].

NK cell activation is partially controlled by KIRs upon binding with their ligands. KIRs constitute a diverse family of activating and inhibitory checkpoint receptors that prevent NK cell activation upon binding with their ligands (primarily human leukocyte antigen-C, HLA-C, molecules) [6]. Distinct KIR family members bind to different major histocompatibility complex (MHC) class I allotypes. The clinical relevance of KIR inhibition has been shown in allogeneic haplo-mismatched stem cell transplantation (alloSCT) in patients with AML. Mismatches between KIRs on donor NK cells and recipient MHC class I molecules enable NK cell activation, which is associated with improved relapse-free survival and overall survival. The results of these stem-cell transplantation studies suggest that, in the absence of KIR interactions with MHC class I molecules, alloreactive NK cells may eradicate residual leukemia [7].

We hypothesized that a pharmacological blockade of KIR–MHC interactions using an anti-KIR monoclonal antibody (mAb) may mimic the KIR mismatch situation in the haplo-alloSCT setting and improve NK cell antileukemic effects. For this purpose, we generated the anti-KIR mAb IPH2101 (formerly called 1-7F9), which specifically binds with high affinity to the inhibitory KIR2DL1, -2 and -3 receptors (and activating KIR2DS1 and -2 receptors) that are expressed on approximately half of peripheral blood NK cells and blocks the interaction with HLA-C. *In vitro*, IPH2101 dose-dependently augments NK cell–mediated killing of autologous human AML blasts that express HLA-C [8]. *In vivo* efficacy was demonstrated in a non-obese diabetic severe combined immunodeficiency (NOD-scid) mouse model of NK cell–mediated tumor rejection [8]. Two phase 1 studies conducted in patients with AML [9] and multiple myeloma [10] have shown that dose-dependent long-term KIR receptor saturation can be achieved *in vivo* in humans, without significant toxicity. On the basis of this encouraging initial data, clinical development of anti-KIR mAbs was pursued.

IPH2101 is a fully human IgG4 that is produced from human hybridoma cells. To increase yield and avoid the formation of half -antibodies described with human IgG4, a recombinant version, lirilumab (IPH2102/BMS-986015/BMS-986015-01), was developed. Lirilumab (IPH2102) is a fully human IgG4 monoclonal antibody produced by recombinant Chinese hamster ovary cells. Lirilumab recognizes the same epitope as IPH2101. It has the same primary amino acid sequence as IPH2101, except for one mutation introduced in the constant region of the heavy chain, where a serine is substituted for a proline. This mutation leads to slight changes in the glycosylation of the antibody. Lirilumab has the same mechanism of action as IPH2101 and therefore similar *in vivo* pharmacologic properties are expected. However, the point mutation in the IgG4 heavy chain could potentially affect the pharmacokinetics and the duration of receptor occupancy.

We present here the results of a first-in-human, phase 1 study of escalating doses of lirilumab in patients. The patients had either solid tumors (carcinomas of the breast [11], kidney [12] or ovaries [13]) or hematologic malignancies (AML [14] or chronic lymphocytic leukemia (CLL) [15]) that are known to be sensitive to NK cell control. The study was designed to assess the safety, pharmacokinetics and pharmacodynamics of lirilumab and to determine the recommended dose for future studies.

## RESULTS

### Patients

Thirty-seven patients were included in the study, and each patient received at least one dose of lirilumab. Twenty patients were included in the dose-escalation phase, and a different 17 in the extension phase. Patient characteristics are detailed in Table 1. The median age of the patients was 62 years. Fifty-nine percent (22/37) of the patients had hematological malignancies, including AML, CLL and indolent NHL (which included lymphocytic, follicular, marginal zone and mantle cell lymphomas and also Waldenstrom dysglobulinemia); 41% (15/37) of the patients had solid tumors (breast, ovarian and 2 others) (Table 1). At baseline, 15 patients were in CR, 20 in PR, and two had slowly progressive disease.

**Table 1:** Patient characteristics

	*N* (%)
Median age (years) (range)	62 (33–73)
Sex (M/F)	14/23
ECOG PS	
0	23 (62%)
1	14 (38%)
Hematological malignancy	22 (59.5%)
AML	5 (22.7%)
CLL	6 (27.3%)
Indolent NHL	11 (50.0%)
Solid tumor	15 (40.5%)
Breast cancer	6 (40.0%)
Ovarian cancer	7 (46.7%)
Other (pancreatic cancer, endometrial cancer)	2 (13.3%)
Prior lines of treatment	
0–1	13 (35%)
2–3	19 (51%)
≥4	5 (14%)
Disease status at baseline	
CR	15 (40.5%)
PR	20 (54.1%)
SPD	2 (5.4%)
Median interval from diagnosis to inclusion in study, months	37 (3.5–315.7)

ECOG PS: Eastern Cooperative Oncology Group performance status; AML: acute myeloid leukemia; CLL: chronic lymphocytic leukemia; NHL: non-Hodgkin lymphoma; CR: complete response; PR: partial response; SPD: slowly progressive disease.

### Safety and tolerance

During the dose-escalation phase, six dose levels were explored (Table 2). No DLT was reported, and the MTD was not reached for doses up to 10 mg/kg.

**Table 2:** Dose-escalation schema and tolerance

Escalation phase				Extension phase		
Dose level (mg/kg)	No. of patients	DLT	Patients with grade 3–4 lirilumab-related adverse events	Dose level (mg/kg)	No. of patients	Patients with grade 3–4 lirilumab-related adverse events
**0.015**	3	0	0	**0.015**	9	5
**0.3**	3	0	0			
**1**	4	0	0			
**3**	4	0	0	**3**	8	2
**6**	3	0	1^a^			
**10**	3	0	0			

^a^Transient and asymptomatic grade 3 elevation of lipase that occurred >1 month after the first administration of lirilumab and thus was not considered to be a DLT.

Seventy percent of patients in both the dose-escalation and extension phases received the four scheduled cycles. In the escalation phase, in which a negative KIR occupancy had to be achieved before proceeding to cycle 2 (up to a dose of 3 mg/kg), the median number of days between cycle 1 day 1 and cycle 2 day 1 was 168 days (range: 27–268 days). In the extension phase, the median interval between cycle 1 day 1 and cycle 2 day 1 was 29 days (range: 28–50 days).

Eighteen patients withdrew from the study early, mainly because of disease progression (13 patients) or because of lirilumab-related adverse events (AEs) (3 patients); the AEs included liver function test (LFT) abnormality, grade 1 papular rash, and grade 3 allergy (one patient each). All AEs were observed in patients treated at the 0.015 mg/kg dose level.

All but one patient (97%) had at least one treatment-emergent AE during the study (Table 3). However, treatment was generally well tolerated, and all AEs were mild and transient. A total of 25 (68%) patients had AEs deemed to be related to lirilumab. AEs evaluated as being related to lirilumab and reported in >10% of patients were pruritus (19%), asthenia (16%), fatigue (14%), infusion-related reaction (14%), and headache (11%). The lirilumab-related AE rates varied across the doses tested but did not show a dose–response relationship. Seven (19%) patients had grade 3 or 4 lirilumab-related AEs (asymptomatic increased serum lipase in 2 patients; decreased lymphocyte count, presyncope, increased serum bilirubin, increased gamma-glutamyl transpeptidase levels, abnormal LFT, urticaria plus hypersensitivity, and angioedema in 1 patient each).

**Table 3:** Adverse events occurring in ≥10% of total patients regardless of causality

	Lirilumab 0.015 mg/kg (*N* = 12)		Lirilumab 0.3 mg/kg (*N* = 3)		Lirilumab 1 mg/kg (*N* = 4)		Lirilumab 3 mg/kg (*N* = 12)		Lirilumab 6 mg/kg (*N* = 3)		Lirilumab 10 mg/kg (*N* = 3)		Total (*N* = 37)	
	All Grades *N* (%)	Grade 3 or 4 *N* (%)	All Grades *N* (%)	Grade 3 or 4 *N* (%)	All Grades *N* (%)	Grade 3 or 4 *N* (%)	All Grades *N* (%)	Grade 3 or 4 *N* (%)	All Grades *N* (%)	Grade 3 or 4 *N* (%)	All Grades *N* (%)	Grade 3 or 4 *N* (%)	All Grades *N* (%)	Grade 3 or 4 *N* (%)
Any AE	11 (92)	7 (58)	3 (100)	2 (67)	4 (100)	3 (75)	12 (100)	9 (75)	3 (100)	2 (67)	3 (100)	2 (67)	36 (97)	25 (68)
Asthenia	4 (33)	0	1 (33)	0	1 (25)	0	5 (42)	0	1 (33)	0	0	0	12 (32)	0
Pruritus	3 (25)	0	1 (33)	0	1 (25)	0	4 (33)	0	0	0	0	0	9 (24)	0
Headache	1 (8)	0	2 (67)	0	1 (25)	0	1 (8)	0	2 (67)	0	1 (33)	0	8 (22)	0
Disease progression	2 (17)	2 (17)	0	0	1 (25)	1 (25)	1 (8)	1 (8)	1 (33)	1 (33)	2 (67)	2 (67)	7 (19)	7 (19)
Fatigue	1 (8)	0	3 (100)	0	1 (25)	0	0	0	0	0	1 (33)	0	6 (16)	0
Hypertension	0	0	1 (33)	0	2 (50)	1 (25)	2 (17)	2 (17)	1 (33)	0	0	0	6 (16)	3 (8)
Constipation	2 (17)	0	0	0	1 (25)	0	0	0	2 (67)^a^	0	0	0	5 (14)^a^	0
Cough	3 (25)	0	0	0	0	0	2 (17)	0	0	0	0	0	5 (14)	0
Diarrhea	2 (17)	0	0	0	0	0	2 (17)	0	1 (33)	0	0	0	5 (14)	0
Hyperuricemia	0	0	1 (33)	1 (33)	0	0	3 (25)	2 (17)	0	0	1 (33)	1 (33)	5 (14)	4 (11)
Infusion-related reaction	2 (17)	0	0	0	0	0	2 (17)	0	0	0	1 (33)	0	5 (14)	0
Nausea	2 (17)	0	0	0	0	0	2 (17)	0	0	0	1 (33)	0	5 (14)	0
Dyspnea	1 (8)	0	0	0	1 (25)	0	1 (8)	0	1 (33)	0	0	0	4 (11)	0
Pyrexia	2 (17)	0	0	0	0	0	1 (8)	0	0	0	1 (33)	0	4 (11)	0
Rash	1 (8)	0	1 (33)	0	0	0	2 (17)	0	0	0	0	0	4 (11)	0

AEs were graded using the Common Terminology Criteria for Adverse Events (CTCAE) severity scale. Only the worst intensity was counted for multiple occurrences of the same adverse event in any one patient.

^a^One patient who experienced constipation >100 days after the last treatment was not included.

No lirilumab-related hematological toxicity was observed. One patient experienced grade 3–4 neutropenia 24 hours after the first and second administrations of 0.015 mg/kg, but this was reversed in a few days, suggesting a transient margination of neutrophils phenomenon.

### Response

Twenty-two of the 37 patients had evaluable disease at baseline: 5 with CLL in PR, 7 with NHL (including 5 in PR and 2 with slowly progressive disease, 5 with breast cancer in PR, 4 with ovarian cancer in PR, and 1 with pancreatic cancer in PR. At the end of study, none of the patients had achieved an objective response: 15 had progression and 22 had stable disease. Notably, one patient with ovarian cancer and peritoneal carcinomatosis in PR remained stable without therapy at 18 months after inclusion. Progression-free survival by diagnosis is described in Table 4.

**Table 4:** Progression-free survival by diagnosis

Type of Cancer	No of patients	Median PFS (months)	Range (months)
Acute myeloid leukemia	5	6.6	1–10.4+
Breast cancer	6	Not reached	1.3–16.5+
Chronic lymphocytic leukemia	6	19.6	3.5–19.5+
Endometrial cancer	1	-	7.7
Indolent non-Hodgkin lymphoma	11	Not reached	3.7–16.1+
Ovarian cancer	7	5.3	3.7–13+
Pancreatic carcinoma	1	-	1.9

PFS: Progression-free survival.

### Pharmacokinetics

**Figure 1:**
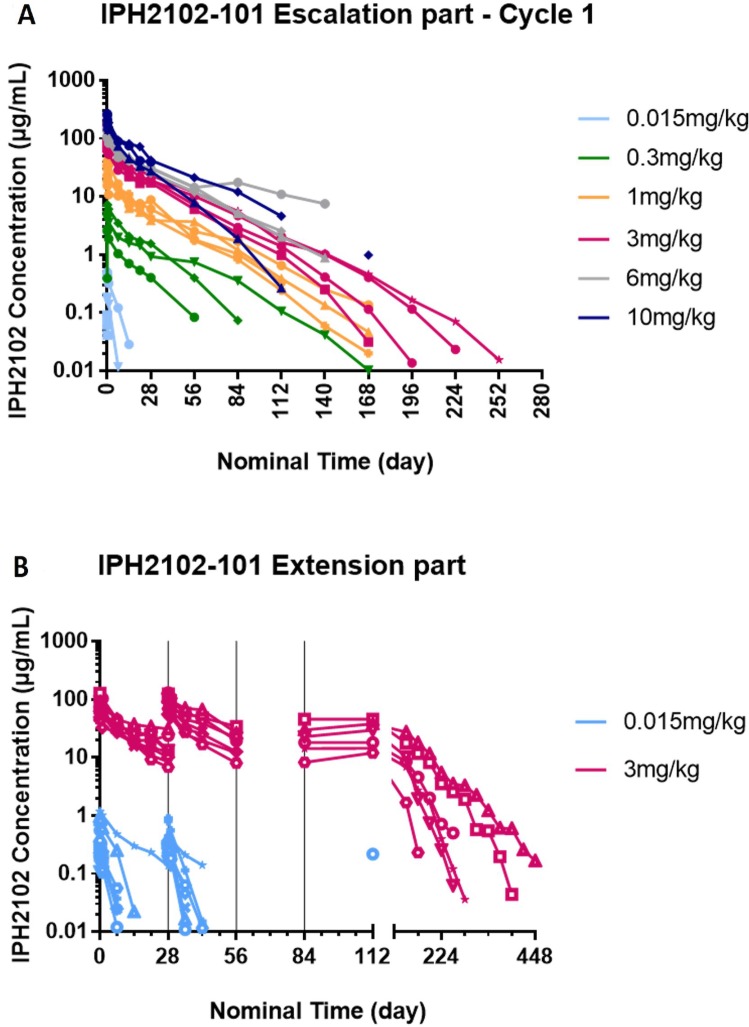
Individual serum concentrations of lirilumab versus nominal time after the first administration of lirilumab (**A**) and after repeated administration of lirilumab in the study extension phase (**B**). (A) after the first administration of lirilumab (cycle 1). (B) after repeated administrations of lirilumab in the study extension phase. In the escalation phase of the study, samples were collected at baseline on day 1, then at 10 min (0.015 mg/kg dose only), 1 h, 3 h, 6 h, day 2, day 8, day 15, day 22, day 29, day 43 (except 0.015 mg/kg dose), and every 4 weeks thereafter until KIR occupancy decreased below 30% (A). In the extension phase, samples were collected at baseline before dosing on day 1, then at 10 min (0.015 mg/kg dose only), 1 h, 3 h, 6 h, day 2, weekly during cycles 1 and 2, then every 4 weeks from day 1 for cycle 3 until the end-of-treatment visit (B). Nominal times are used. Lower limits of quantification (LLOQ) = 10 ng/mL. Serum concentrations below LLOQ are not included in the profiles. Each color represents a dose cohort and each symbol represents individual patient data in each cohort.

Figure 1A shows individual serum concentrations of lirilumab versus nominal time following the first lirilumab administration in the dose-escalation phase of the study. Mean concentration levels declined in a bi-exponential manner after the end of the infusion. These results suggested dose dependency and a linear pharmacokinetic profile that is typical for therapeutic antibodies. Figure 1B shows individual serum concentrations of lirilumab versus nominal time in the extension phase of the study. These results suggested no accumulation of lirilumab with 4-weekly repeated administrations.

The initial pharmacokinetic model that was developed and validated for lirilumab involved two compartments for disposition and parallel linear and saturable elimination pathways, which were predicted to be negligible at concentrations of 3 µg/mL and above. The lirilumab pharmacokinetic parameters obtained in the final structural pharmacokinetic model were: central volume of distribution (in men) 3.95 L; clearance (in a 70-kg patient with 2.8% KIR on T cells at baseline) 0.173 L/day; peripheral volume of distribution 3.07 L; inter-compartmental clearance 0.620 L/day; V_max_ 64.0 µg/day; and K_m_ 268 ng/mL. Formal stepwise exploratory covariates ultimately retained the effects of the baseline percentage of KIR on T cells (power model, 2.4-fold clearance variation within the range of observed baseline %KIR T cells values) and body weight on clearance (power model with an exponent of 0.80), as well as the effect of gender on central volume of distribution (68.9% of men in women).

The final pharmacokinetic model was used to simulate individual single-dose pharmacokinetic profiles that were subjected to a noncompartmental analysis to derive exposure parameters; the results are presented in Table 5. There was a dose-proportional increase in maximum concentration and Exposure (Area under the curve) at dose levels above 0.3 mg/kg. This suggests a linear PK for lirilumab at such doses, with negligible impact of target mediated drug disposition.

**Table 5:** Summary statistics on exposure metrics stratified by dose level

		Geometric Mean (CV%)					
Dose level	(mg/kg)	0.015	0.3	1	3	6	10
*N*		12	3	4	12	3	3
C_max_	(ng/mL)	297 (50.9)	5,722 (35.7)	24,650 (20.4)	76,126 (25.7)	152,872 (7.51)	197,890 (7.02)
C_max_/dose	(ng/mL)/µg	0.251 (52.5)	0.334 (33.1)	0.384 (21.6)	0.345 (25.0)	0.42(5.78)	0.290 (8.28)
C_min (28 days)_	(ng/mL)	8.56 (370)	972 (65.6)	5615 (9.79)	13,935 (31.9)	29,810 (19.4)	39,455 (36.3)
AUC_0–28_	(ng·day/mL)	1519 (70.2)	49,240 (47.4)	263,355 (11.8)	719,907 (20.8)	1,347,718 (16.7)	2,006,586 (12.2)
AUC_0–28_ /dose	(ng·day/mL)/µg	1.28 (78.4)	2.88 (28.8)	4.10 (11.6)	3.26 (20.7)	3.74 (11.0)	2.94 18.0)

N: Number of patients; CV%: coefficient of variation of the geometric mean; C_max_: maximum concentration; C_min_ (28 days): trough concentration at day 28; AUC_0–28_: AUC single dose between 0 and day 28; Dose: actual dose in µg.

### Immunogenicity evaluation

Three of the 37 patients had measurable HAHA at baseline at very low titers (titer = 1, as determined by serial dilution in titration assays), and one patient (in the 0.015 mg/kg level of the extension phase of the study) developed low titer (<3) of HAHA during the course of the study. However, the presence of HAHA at baseline did not seem to have any impact on the pharmacokinetic profile of lirilumab. In conclusion, lirilumab is not immunogenic.

### Pharmacodynamics

**Figure 2:**
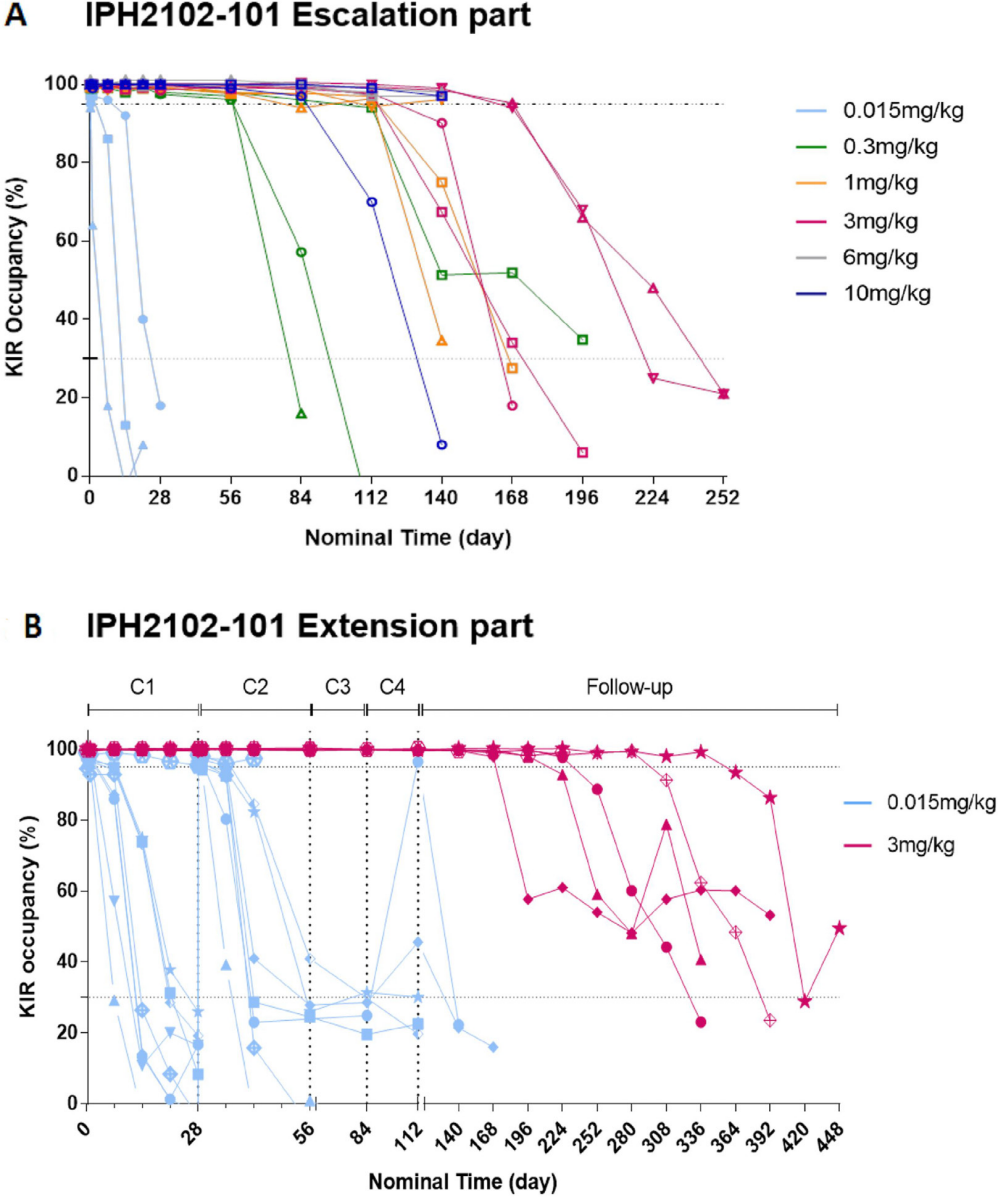
Individual KIR saturation versus nominal time after the first administration of lirilumab (cycle 1) in the dose-escalation phase (**A**) and after repeated administration of lirilumab in the extension phase (**B**). A. after the first administration of lirilumab (cycle 1) in the dose-escalation phase. (A) PE-conjugated lirilumab was used to assess free KIR2Ds on peripheral blood NK cells, which gave an estimate of KIR occupancy. Cells with free KIR2Ds were defined as CD3^–^ CD16/CD56^+^ lymphocytes. During the dose-escalation phase, assessments were performed on cycle 1 day 1 (at baseline, 3 h and 24 h), then weekly during the first 4 weeks, and then every 4 weeks (until KIR occupancy was <30% or up to 6 months for doses above 3 mg/kg). Each color represents a dose cohort and each symbol represents individual patient data in each cohort. (B) after repeated administration of lirilumab in the extension phase. KIR occupancy by lirilumab was assessed on cycles 1 and 2 day 1 (baseline, 3 h, 24 h), then weekly until the end of cycle 1 until day 15 of cycle 2; on cycles 3 and 4 day 1 (0 h, 3 h); and every 4 weeks. The KIR saturation curves for individual patients treated with lirilumab at doses of 0.015 mg/kg (blue line) and 3 mg/kg are shown (red line). Each color represents a dose cohort and each symbol represents individual patient data in each cohort.

In the dose-escalation phase of the study, after the first lirilumab administration, a high level of KIR occupancy (>95%) was reached for all doses in all patients (see Figure 2A), with the exception of one patient (patient 0202), who received 0.015 mg/kg and who had abnormally high numbers of peripheral NK and KIR^+^ NK cells (1113 KIR^+^ NK cells/µL; the levels in the other patients ranged from 18 to 378 NK cells/µL (mean, 93 NK cells/µL). A KIR occupancy >95% was observed for at least 24 hours but for less than 7 days at a dose of 0.015 mg/kg, for at least 2 months and less than 3 months at a dose of 0.3 mg/kg, and for at least 4 months at a dose of 1 mg/kg.

As shown in Figure 2B, 4-weekly administration of 0.015 mg/kg lirilumab in the extension phase allowed intermittent high KIR occupancy (>95% for between 3 hours and 7 days, then decreasing) in all but one (patient 0212) patients. Moreover, 4-weekly administration of 3 mg/kg lirilumab allowed sustained high KIR occupancy (>99%) between administrations, with a high KIR occupancy (>98%) maintained for 2–8 months after the end-of-treatment visit. Similar results of saturation were obtained using antibodies specific to KIR2DL1 or KIRDL2/L3/S2 for detection of free receptors or by the detection of the lirilumab bound to peripheral NK cells (data not shown).

### Immuno-monitoring

**Figure 3:**
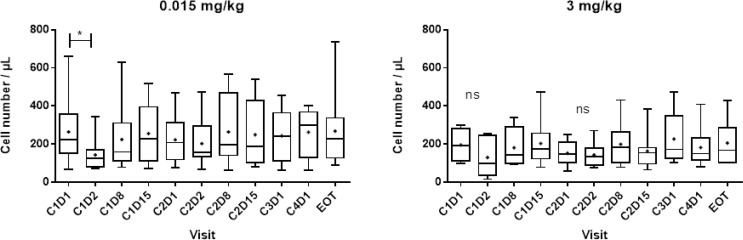
Number of peripheral blood NK cells after repeated administration of lirilumab Absolute cell numbers of the peripheral blood lymphocyte subsets were assessed by flow cytometry (TruCount™ beads; Becton Dickinson). C: cycle; D: day; EOT; end-of-treatment visit. Box (25th to 75th percentiles and median) and whiskers (minimum to maximum) plots are shown. High inter-patient variability was observed in the cohort that received 0.015 mg/kg, but this was independent of the treatment. As observed previously with IPH2101, a transient decrease in the peripheral NK cell number was detected following the first administration of lirilumab. This observation was not dependent on the dose.

**Figure 4:**
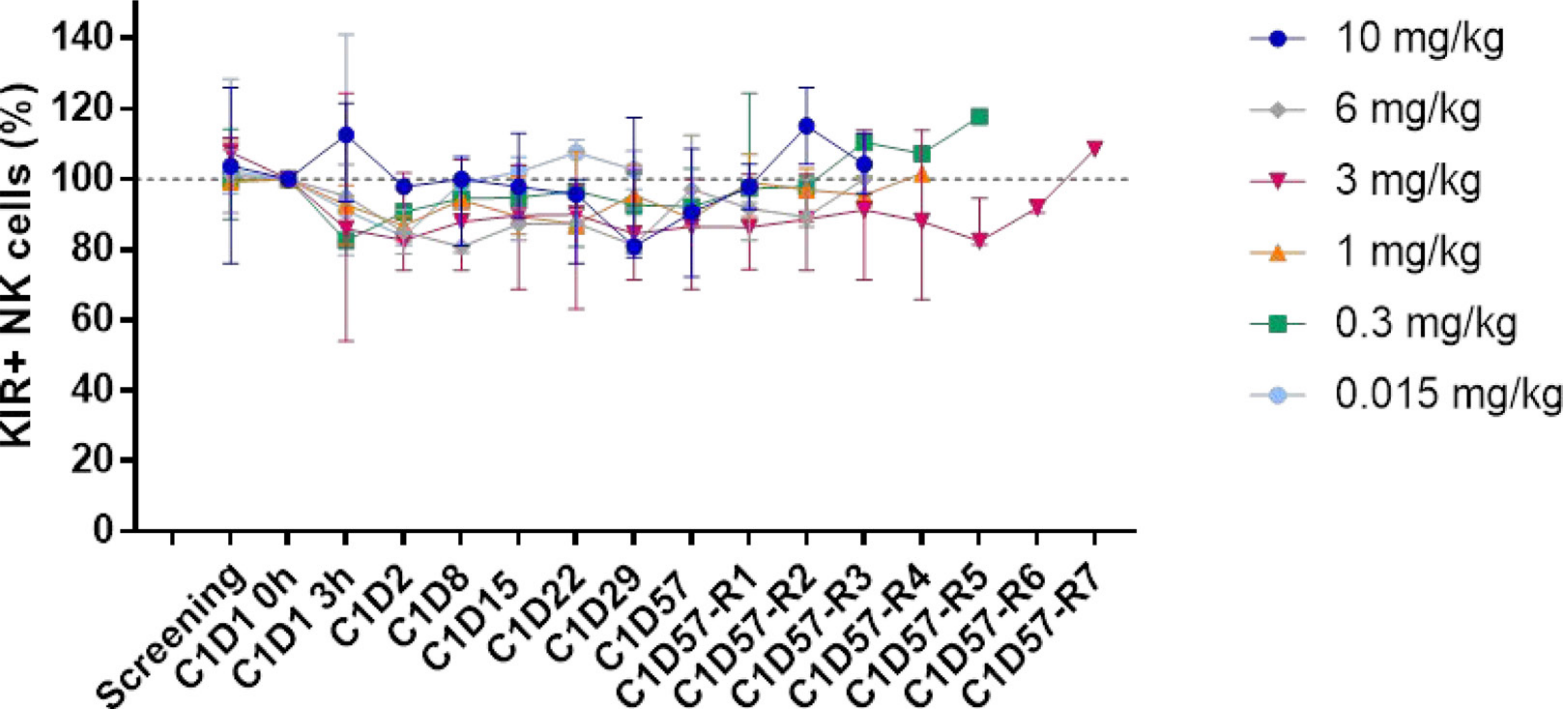
Distribution of peripheral KIR2D+ NK cells after lirilumab administration The distribution of KIR^+^ NK cells was assessed on whole blood by flow cytometry at the indicated visit (for up to 252 days/36 weeks depending on the dose and the patient). KIR^+^ cells were identified by staining with lirilumab followed by PE-conjugated mouse anti-human IgG4 (HP6025);. For each patient, the baseline (cycle 1 day 1 (C1D1) 0 h) value was set as 100% and the ratio of the value obtained at each visit to the baseline value was calculated. The median and the range for each cohort are shown. C1D57R = C1D57 repeat visit (1 to 7) every 4 weeks.

Decreases in NK cell absolute numbers and percentages were observed at cycle 1 day 2, but they rapidly recovered (Figure 3). No other significant change in the number or the distribution of lymphocyte subpopulations targeted by lirilumab was observed, even though full KIR occupancy was observed for at least 3 months (with doses >3 mg/kg) (Figure 4).

**Figure 5:**
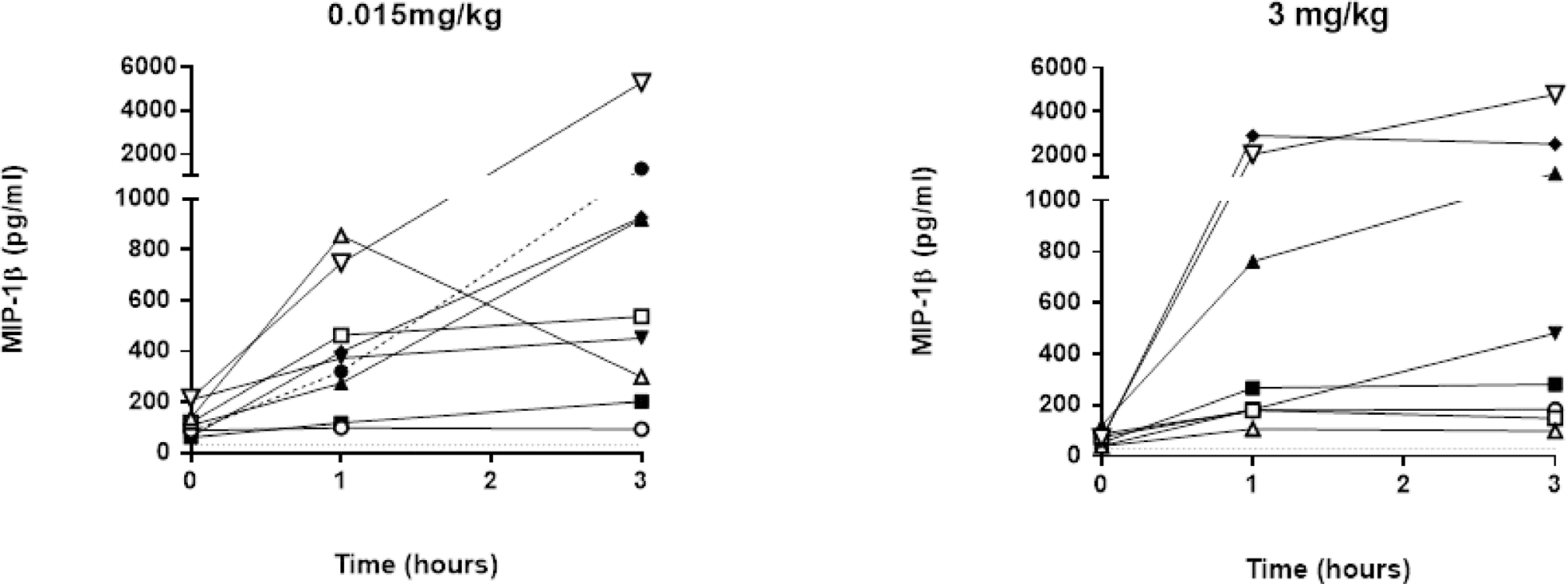
MIP-1β concentration after lirilumab administration The MIP-1β concentration (Lower limit of quantification (LLOQ) = 30 pg/mL) was assessed on plasma samples collected in the extension phase of the study at–baseline (0), 1 h, 2 h and 3 h post-lirilumab infusion in cycle (C) 1. Lower limits of quantification (LLOQ) = 30 ng/mL. Each symbol represents individual plasma concentrations across time and in each dose cohort.

No significant changes in IL1-β, IL6, or IFN-γ production were observed following lirilumab administration (data not shown); this is consistent with an absence of major clinical cytokine release syndrome. Interestingly, TNF-α and macrophage inflammatory protein (MIP)-1β (Figure 5) increased 1–3 hours after the first administration of lirilumab, regardless of the dose. However, only limited secretion of these cytokines was observed after the second administration of 0.015 mg/kg of lirilumab, and no secretion was observed after the second administration of 3 mg/kg (see Figure 5). Presumably, this lack of cytokine secretion following the second administration was due to the presence of already high levels of antibody in the circulation from the initial administration. Finally, no significant change in CD69 expression on KIR-positive NK cells was observed as previously described following IPH2101 administration [9].

## DISCUSSION

In previous reports, we [9] and others [10] have shown that blockade of inhibitory KIRs could be achieved *in vivo* using the IPH2101 anti-KIR mAb. In patients with AML in CR or with myeloma, long-term (i.e., >4 weeks) full KIR saturation was achieved without significant clinical or biological side effects with doses up to 3 mg/kg. In the present study, our goal was to evaluate the safety and tolerability of escalating doses of lirilumab, a fully human IgG4 mAb, and to determine the optimal dose and schedule for future phase 2 studies.

Our results demonstrate that lirilumab treatment was well tolerated. Patients with various hematological or solid malignancies experienced only mild and transient side effects, mainly rash, infusion reactions, and headache. The MTD was not reached for doses up to 10 mg/kg.

The pharmacokinetic characteristics were as expected for an IgG mAb, with dose-dependent serum concentrations that declined in a bi-exponential manner, and no marked accumulation following repeated dosing. In addition, lirilumab was not immunogenic (only one patient developed low-titer HAHA during the course of treatment, with no impact on pharmacokinetic parameters).

With lirilumab (as with IPH2101), full KIR saturation was achieved, and its duration was dose-dependent. Although the structural differences between the two compounds rely on a single amino-acid difference, substantial pharmacodynamic differences were observed. Indeed, with repeated 4-weekly administration, full saturation (>95% KIR occupancy) for more than 4 weeks was achieved with lirilumab at doses ≥0.3 mg/kg, whereas the same was achieved with IPH2101 only at doses of 3 mg/kg. Similarly, the KIR occupancy duration was dose-dependent and lasted more than 4 months for doses of 1 mg/kg and above. This was associated with limited immunological modifications of lymphocyte subsets.

Absent or low cytokine release was observed, consistent with the good clinical tolerance to the treatment. No signs of NK-cell activation, studied using CD69 expression were recorded, confirming the selectivity of lirilumab effects. This allows us to rule out a major a priori concern that the blockade of molecules that inhibit NK cells might lower the activation threshold against normal cells that may express low levels of activation ligands or may directly trigger KIR2DL1/S1 and KIR2LDL3/S2 on NK cells, which could have represented a safety issue.

In addition, lirilumab administration had no deleterious impact on the number of circulating NK cells, specifically in the KIR2D-positive NK-cell compartment. A recent report suggested that, in patients with refractory/relapsed myeloma, treatment with the original anti-KIR antibody IPH2101 resulted in reduced KIR2D expression on NK cells and impaired *ex vivo* NK cell responsiveness [16]. The authors proposed that this may be explained by the removal of KIR2D from the surface of NK cells by trogocytosis. Such phenomenon was not observed in our study. This difference may be explained by different *ex vivo* processing. In the present study, KIR2D expression was monitored in whole blood samples with normal levels of endogenous human IgG, allowing the continuous saturation of the Fc receptors (i.e., CD64) and preventing the potential trogocytosis-induced KIR2D down-modulation described by others.

During the extension phase of this study, we were able to show that repeated doses of 0.015 mg/kg lirilumab every 4 weeks led to intermittent full blockade of KIRs and that doses of 3 mg/kg led to continuous blockade. *In vitro* experiments and *in vivo* tumor rejection models indicate that full KIR occupancy is needed to achieve optimal enhancement of NK cell activity and, therefore, optimal clinical efficiency.

However, some preclinical data suggest that transient full occupancy may be optimal for long-term treatment. Prolonged KIR inhibition may negatively affect NK cell education, especially over long periods of treatment with lirilumab. During their development, NK cells acquire functional competence in a process referred to as “education”. It has been shown in both mice [17, 18] and humans [19, 20] that this acquisition of functional NK cell competence relies on interactions between KIRs (or their equivalent in mice) and cognate ligands. Conceivably, continuous full KIR blockade over time may impede the development of new, functionally competent NK cells. Conversely, transient KIR blockade might allow both the optimal activation of NK cells for 1–2 weeks following each administration of the antibody and the education of new NK cells towards the end of each treatment cycle when occupancy is reduced. Therefore the optimal dosing scheme could possibly aim for intermittent full KIR occupancy. This study establishes that both continuous and intermittent KIR occupancy are safe in human cancer patients and provides a basis for testing both types of schedules in a randomized phase 2 clinical trial in patients with AML [21].

The mechanism of action of lirilumab paves the way for therapeutic combinations of drugs that modulate other immunoregulatory mechanisms and/or other immune effector cells. These include mAbs that block CTLA-4 or PD-1 checkpoints [1]. Indeed, KIR and CTLA-4 or KIR and PD-1 regulate nonredundant inhibitory pathways and can be expressed by distinct immune effectors (mainly CD8+ T cells for PD-1 and NK cells for KIR2D). Furthermore, blockade of CTLA-4 or PD-1 on T cells can induce the release of cytokines, including IL-2, that enhance NK cell function, whereas blockade of KIR can result in the secretion of IFN-γ that may boost both myeloid- and T cell-mediated antitumor responses [22]. Therefore, combination treatment with checkpoint blockers that stimulate T cells and NK cells may promote NK–T-cell cross-talks, leading to more robust responses in some patients who fail to respond to treatment with single agents. Encouraging preliminary safety and efficacy data from phase 1 or phase 2 trials of the combination of lirilumab with nivolumab and/or ipilimumab (NCT01714739/NCT01750580) have been recently presented [23, 24].

Through enhancement of NK cell activity, KIR2D blockade may also improve antibody-dependent cellular cytotoxicity. Therefore, lirilumab may act synergistically with other mAbs; such synergism has been demonstrated between lirilumab and the anti-CD20 mAb in an animal model [25].

In addition, hypomethylating drugs (5-azacytidine or decitabine) that are currently approved as treatments for AML and MDS have shown immunomodulatory effects, including enhanced production of Th1-type chemokines [26] and up-regulation of NKG2D ligand (MICA/B, ULBP) expression [27, 28]. Lirilumab in combination with 5-azacytidine is currently being assessed as a treatment for relapsed/refractory AML (NCT02399917) and MDS (NCT02599649).

Finally, a combination of lirilumab with immunomodulatory drugs might also be considered. The latter therapeutic class, besides having direct antitumor effects, increases the production of various cytokines, including IFN-γ and IL-2, and enhances the functions of NK and T cells [29]. The combination of IPH2101 with the immunomodulatory drug lenalidomide enhances the cytotoxicity of NK cells against autologous myeloma *in vitro* [30] and has been safely administered to patients with relapsed/refractory multiple myeloma [10].

In conclusion, this study showed that prolonged KIR blockade using lirilumab is safe and well tolerated in patients with various types of cancers (hematological malignancies or solid tumors). We identified the doses that are able to produce full KIR occupancy without deleterious clinical, hematological, or immunological effects, thus confirming the selectivity of this approach. These findings support the rationale for the choice of dose and schedule in several clinical trials of lirilumab given in monotherapy or in combination with various immunotherapeutic or immunomodulatory agents.

## MATERIALS AND METHODS

### Patient selection

Patients with various malignancies were eligible, including those with AML in first or second complete response (CR/CRi), excluding acute promyelocytic leukemia (APL) and core-binding factor AMLs; CLL in partial response (PR), CR [31], or slowly progressing and not requiring (or not eligible for) standard therapy; indolent non-Hodgkin lymphoma (NHL), including mantle-cell lymphoma, in PR, CR [32], or slowly progressing and not requiring (or not eligible for) standard therapy; stage IV solid tumor in CR, PR, or slowly progressing after standard therapy. In addition, to be eligible for the study, patients had to have Eastern Cooperative Oncology Group performance status (ECOG PS) of <3; age between 18 and 80 years; adequate renal and hepatic functions; absolute neutrophil count >1 × 10^9^/L; platelet count >75 × 10^9^/L; no treatment with immunotherapy, lymphoablative chemotherapy, monoclonal antibodies, high-dose chemotherapy with autologous hematopoietic-cell transplantation or G-CSF 28 days or less prior to inclusion; no prior history of active autoimmune disease or monoclonal gammopathy; and no active infectious disease.

All patients provided written informed consent. The study protocol was approved by the CPP Sud Méditérranée I ethics committee and was registered as EUDRACT N° 2009-011526-33.

### Treatment and study design

Lirilumab (IPH2102) was provided by Innate Pharma (Marseille, France) as 10 mg/mL vials. It was administered intravenously, at six dose levels (0.015, 0.3, 1, 3, 6 and 10 mg/kg). A classical 3 + 3 phase I design was used. Briefly, consecutive cohorts of 3 patients received escalating doses of lirilumab until the maximum tolerated dose (MTD) was reached or until the maximal dose of 10 mg/kg in the absence of dose-limiting toxicity (DLT). If one patient presented dose-limiting toxicity in a given cohort, that cohort was to be expanded to a total of 6 patients. DLT was defined as the occurrence of CTCAE grade 3 or 4 adverse event during the first month and that was considered by the investigator to be at least possibly related to the administration of lirilumab. The MTD was defined as the highest dose level at which a maximum of 1 out of 6 patients had DLT.

Patients were scheduled to receive 4 cycles of treatment. During the dose-escalation phase of the trial and for safety evaluation, the patients treated at the first four dose levels (0.015, 0.3, 1, and 3 mg/kg) could not proceed to cycle 2 until the KIR occupancy reached ≤30% and not earlier than 28 days after the first administration . However, for doses above 3 mg/kg, because of the long-lasting KIR occupancy, the second cycle was administered 6 months after the first administration regardless of residual receptor occupancy. The subsequent cycles were administered every 28 days, also irrespective of KIR occupancy. All patients were followed up after the end of treatment until KIR occupancy reached ≤30% for safety evaluation.

After completion of the dose-escalation phase of the study, an extension phase was initiated in which patients were allocated to two dose levels: a low dose of 0.015 mg/kg, resulting in full KIR occupancy lasting up to 21 days (i.e., transient KIR occupancy), and a high dose of 3 mg/kg, resulting in prolonged, full KIR occupancy lasting for >28 days (continuous KIR occupancy). This allowed the comparison of intermittent (low-dose) versus continuous (high-dose) KIR blockade during repeated lirilumab administration in 4-week cycles. In this extension phase, the patients were scheduled to receive 4 cycles every 4 weeks, regardless of the dose level. The primary endpoint was safety and the secondary endpoints included pharmacokinetic and pharmacodynamic assessments.

### Assessment of pharmacokinetics

Pharmacokinetic assessments were performed by ELISA on serum samples collected at baseline and at various time points after lirilumab administration.

The population pharmacokinetic analysis was performed by modeling lirilumab concentration data using nonlinear mixed-effect modeling with Phoenix^®^ WinNonLin6.4, NLME™ 1.3 software. Any lirilumab concentration below the lower limit of quantitation (LLOQ) was not considered in the analysis. A preliminary base population pharmacokinetic model was developed and evaluated for lirilumab in patients from this study to derive major pharmacokinetic parameters that describe lirilumab pharmacokinetics, including: central volume of distribution, clearance, peripheral volume of distribution, inter-compartmental clearance, and the Michaelis-Menten parameters of the saturable clearance pathway, V_max_ and K_m_. Parallel linear and saturable elimination is an accepted approximation for target-mediated drug disposition [33, 34].

An exploratory graphical covariate analysis was then performed to obtain initial insights into the major sources of pharmacokinetic variability. The covariates explored in the analysis were: the nature of the disease; tumor type; body weight; height; body mass index; body surface area; sex; age; serum creatinine; calculated (Cockcroft-Gault) creatinine clearance; glomerular filtration rate (estimated by the Modified Diet in Renal Disease Study equation); pharmacological biomarkers (baseline T cell and NK cell number, and baseline percentage of KIR on T cells and NK cells); presence of human antihuman antibodies (HAHA) in at least one pharmacokinetic sample; and batch number of the injected drug. The covariates identified as significant by the exploratory graphical covariate screening were included in the base population pharmacokinetic model to develop a final population pharmacokinetic model for lirilumab, using the stepwise covariate search method.

Once the final model was successfully evaluated and qualified, final individual pharmacokinetic parameters and dose information for the first administration were used to simulate a single-dose pharmacokinetic profile over 28 days, with a granularity of 0.1 day. Simulated individual pharmacokinetic profiles were submitted to a noncompartmental analysis using Phoenix^®^ WinNonlin 6.4 to derive the following exposure parameters: Maximum Concentration (C_max)_, dose-normalized C_max_, Minimum Concentration (C_min)_ at day 28, Area Under the Curve from day 0 to day 28 (AUC_0–28)_, and dose-normalized AUC_0–28_. Descriptive statistics were derived and stratified by dose level.

### Assessment of pharmacodynamics

Absolute cell numbers of peripheral blood leukocyte subsets, as well as the expression of NK- and T-cell activation markers were assessed by flow cytometry on whole blood collected on EDTA tube with TruCount^™^ beads (Becton Dickinson) and conjugated antibodies (Beckman Coulter and Becton Dickinson).

The saturation of the KIRs targeted by lirilumab expressed on peripheral blood NK cells was evaluated by flow cytometry on whole blood samples after staining with phycoerythrin (PE)-conjugated lirilumab, a method that enables detection of free KIRs. NK cells were defined as CD3^–^ CD16^+^CD56^+^ lymphocytes Quantum TM R-PE MESF beads were used for the quantification of PE-conjugated antibodies. KIR occupancy at any given time point was expressed as the ratio of the fluorescence intensity at that time point to the intensity before drug administration. Values above 95% were considered to correspond to full saturation. Analysis was performed with a FACS CANTO II (Becton Dickinson). Samples were taken at baseline and at various time points after treatment until negative KIR occupancy (i.e., <30%).

Cytokine concentrations of plasma samples collected in the dose-escalation phase of the study were assessed at baseline and at various post-dosing time points. Validated methods involving electrochemiluminescence technology were used to quantify cytokines: for interferon (IFN)-γ, tumor necrosis factor (TNF)-α, interleukin (IL)-6 and IL-1β concentrations, the MSD human proinflammatory-I (4-plex) kit (with an LLOQ of 6 pg/mL for IFN-γ and 3 pg/mL for the other cytokines); and for MIP-1β, the MSD human MIP-1β kit (with an LLOQ of 30 pg/mL).

### Toxicity and response evaluation

Safety was assessed using the Common Terminology Criteria for Adverse Events (CTCAE) version 4.03. Response was assessed using the criteria of Cheson *et al.* for AML, myelodysplastic syndrome (MDS), and NHL [35–37]. The Response Evaluation Criteria in Solid Tumors (RECIST) were used to evaluate the response of solid tumors [38] and CLL [31].

### Statistical methods

Statistical analyses were performed with SAS^®^ version 9.1.3. Disease status, progression-free survival and overall survival were analyzed in a descriptive way.

## Abbreviations

AE: Adverse event
ALLOSCT: Allogeneic hematopoietic stem cell transplantation
AML: Acute Myeloid Leukaemia
CLL: Acute Myeloid Leukaemia
CR: Non Hodgkin Lymphoma
CTLA: cytotoxic T-lymphocyte-associated protein
DLT: Dose limiting toxicity
HAHA: Human Anti-Human antibody
HLA: Human leukocyte antigen
IFN: Interferon
IGG: immunoglobulin G
IL: Interleukin
KIR: Killer Immunoglobuline like Receptors
LFT: Liver function test
MABS: Monoclonal antibody
MHC: Major Histocompatibility Complex
MIP: Macrophage Inflammatory Proteins
MTD: Maximum tolerated dose
NHL: Non Hodgkin Lymphoma
NK: Natural killer cells
PD: Pharmacodynamic
PK: Pharmacokinetics
PR: Partial response
TNF: Tumour Necrosis Factor
